# The Impact of Physician Race and Sex on Patient Ranking of Physician Competence and Perception of Leadership Ability

**DOI:** 10.7759/cureus.34778

**Published:** 2023-02-08

**Authors:** Lauren K Dunn, Elizabeth Pham, Emmad Kabil, Amanda M Kleiman, Ebony J Hilton, Genevieve R Lyons, Jennie Z Ma, Edward C Nemergut, Katherine T Forkin

**Affiliations:** 1 Anesthesiology, University of Virginia School of Medicine, Charlottesville, USA; 2 Anesthesiology, Johns Hopkins University School of Medicine, Baltimore, USA; 3 Public Health Sciences, University of Virginia School of Medicine, Charlottesville, USA; 4 Anesthesiology, West Virginia University School of Medicine, Morgantown, USA

**Keywords:** racial disparity, social desirability bias, sex/gender, race/ethnicity, physician competence, patient perception, bias in medicine

## Abstract

Background

Biases affect patient perceptions of their physician and influence the physician-patient relationship. While racial disparities in care and inequities in the healthcare workforce are well-documented, the impact of physician race on patient perceptions remains unclear. We aimed to investigate the association of physician race and sex on patient perceptions during simulated preoperative encounters.

Methods

Three hundred patients recruited consecutively in the Preanesthesia Evaluation and Testing Center viewed pictures of 4 anesthesiologists (black male, white male, black female, white female) in random order while listening to a set of paired audio recordings describing general anesthesia. Participants ranked each anesthesiologist on confidence, intelligence, and likelihood of choosing the anesthesiologist to care for their family member, and chose the one anesthesiologist most like a leader.

Results

Compared to white anesthesiologists, black anesthesiologists had greater odds of being ranked more confident (OR, 1.45; 95% CI, 1.10 to 1.89; *P*=0.008) and being considered a leader (OR, 2.06; 95% CI, 1.50 to 2.84; *P*<0.0001). Among white participants, black anesthesiologists had greater odds of being ranked more intelligent (OR, 2.08; 95% CI, 1.54 to 2.81; *P*<0.0001) and were more likely to be chosen to care for a family member (OR, 2.26; 95% CI, 1.66 to 3.08; *P*<0.0001). Female anesthesiologists had greater odds of being ranked more intelligent (OR, 1.36; 95% CI, 1.08 to 1.71; *P*=0.009) and were more likely to be chosen to care for a family member (OR, 1.58; 95% CI, 1.27 to 1.97; *P*<0.001) compared with male anesthesiologists.

Conclusions

Contrary to our hypothesis, patients ranked black physicians more highly on multiple competence and leadership quality metrics. Our data likely highlight the role social desirability bias may play in studies of racial disparities within medicine.

## Introduction

Patient perceptions of their physicians have been shown to influence the physician-patient relationship and affect healthcare outcomes [1,2]. Perceived physician cultural competency, for instance, has been associated with improved compliance with medical recommendations and increased satisfaction [3,4]. A patient’s perception may also be impacted by their physician’s race. Patients may be more likely to rate the quality of their care lower if their provider is racially discordant with themselves [5]. Conversely, patients who perceive their physicians as sharing similar characteristics, including race, are more likely to trust their physician and report higher satisfaction scores [2]. However, results on the effect of physicians’ race on patients’ perceptions have been mixed. A recent study using a simulated physician encounter found no difference between participant confidence levels in the white male, white female, black female, or black male physicians [6]. As racism, unfortunately, remains pervasive not only in society but also within medicine, patient perception of physician competence based on race requires investigation.

Perioperatively, the physician-patient relationship dynamics are particularly important for anesthesiologists who have a limited period to gain a patient’s trust and reduce anxiety before surgery. Anxiety may contribute to worse perioperative outcomes such as nerve block failure [7] and worse postoperative pain [8]. Understanding factors that positively or negatively influence patient perceptions of anesthesiologists could aid in strengthening the patient-anesthesiologist relationship and improve patient care and outcomes. We previously demonstrated that factors including body language, age, and sex influence patient perceptions of anesthesiologist confidence, intelligence, and leadership ability [9,10].

The aim of this study was to investigate the association of physician race and sex on patient perceptions during simulated preoperative encounters and to determine whether patient perceptions differ when presented with a white anesthesiologist versus a black anesthesiologist. We hypothesized that patient perceptions of physician competence are influenced by visual bias such that patients would perceive white anesthesiologists as more confident, more intelligent, more like a leader, and would be more likely to choose them to care for a family member when compared to black anesthesiologists. Based on prior results [10], we also hypothesized that patients would continue to display a preference for female anesthesiologists on the measures of confidence and likelihood of choosing the anesthesiologist to care for their family member compared to male anesthesiologists regardless of the physician’s race.

Portions of this work were presented previously as a poster at the annual Association of University Anesthesiologists and International Anesthesia Research Society meetings held virtually on March 18, 2022, and March 20, 2022, respectively.

## Materials and methods

After approval by the institutional review board (University of Virginia Health System, Charlottesville, Virginia), 300 consecutive patients presenting to the University of Virginia Health Preanesthesia Evaluation and Testing Center who met inclusion criteria were enrolled in the study between February 8, 2021, and June 22, 2021. Patients were excluded if they were <18 years old and/or did not speak English. Patients enrolled and participated in the study prior to their consultation with the nurse or anesthesiologist. The institutional review board waived the requirement for written informed consent because no patient identifiers were collected. Participants reviewed the study information letter before verbally consenting to participate. This cross-sectional study adheres to the Strengthening the Reporting of Observational Studies in Epidemiology (STROBE) guidelines.

**Figure 1:**
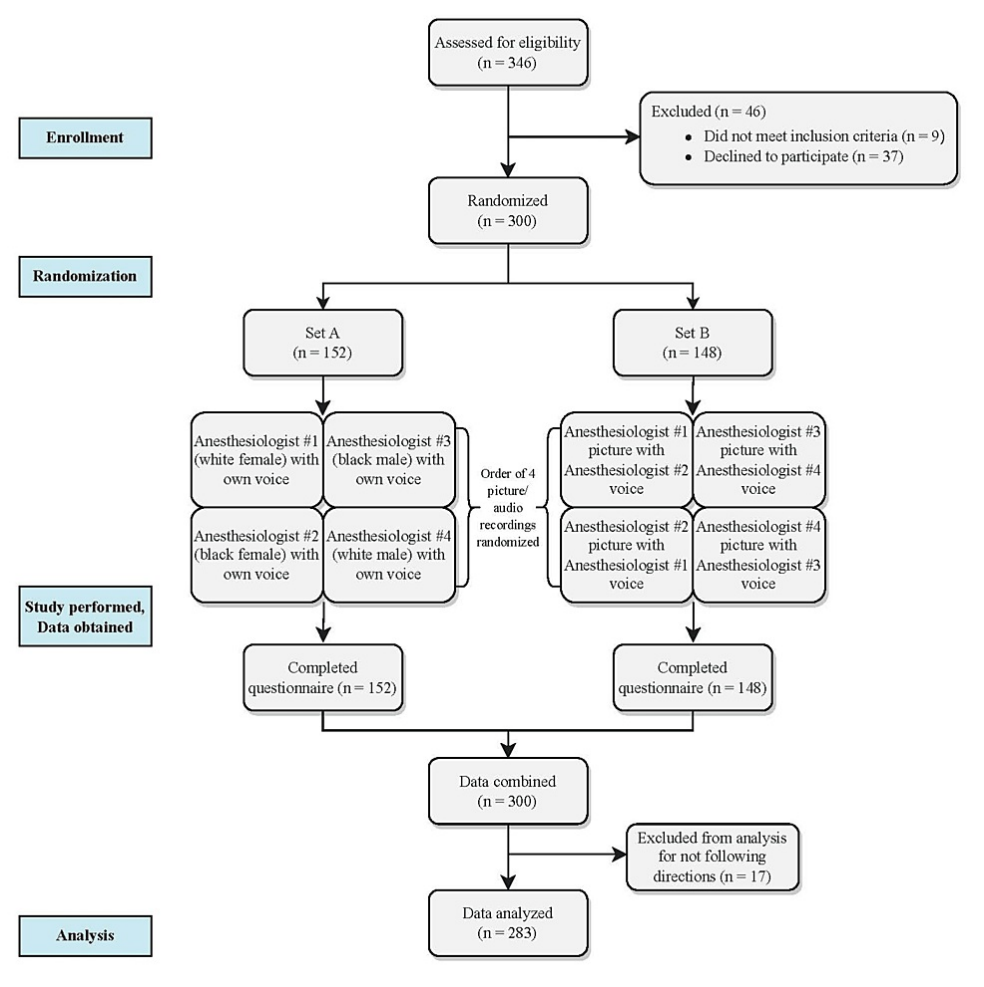
Study Design This Consolidated Standards of Reporting Trials (CONSORT) flow diagram illustrates the organization of the study, including enrollment, randomization, and data analysis.

Figure 1 illustrates the organization of the study. After obtaining verbal consent, each participant viewed four separate image and audio sets of actor anesthesiologists in a quiet room in the Preanesthesia Evaluation and Testing Center. Each image of an actor anesthesiologist was shown for approximately 90 seconds with an associated audio recording of the actor anesthesiologist describing general anesthesia and associated risks using a standardized script. The complete image/audio set was comprised of one black male, one black female, one white male, and one white female actor anesthesiologist displayed in random order. Each actor anesthesiologist displayed the same posture and wore gray scrubs and a white coat. One image/audio set (Set A) was created with the actors’ voices matched to their picture. A second image/audio set (Set B) was created with the voices of the two male actors and the voices of the two female actors switched to play over the picture of the opposing actor. This second set was created to control for any differences in voice tone, tempo, or other unmeasured voice characteristics. We recruited actors that were all approximately the same age (all in their 30s). The comparator racial groups were chosen because black patients are the most prevalent under-represented racial minority in our patient population.

Participants were randomized to view either Set A or Set B and each image/audio pair within the set was displayed in random order. A random number generator (Random.org) was used to generate a list of 300 integers between 1 and 2 to determine which set of picture/audio recordings participants would view (Set A or Set B). A second list of 300 sequences of integers between 1 and 4 was created to assign the order in which participants viewed each picture/audio recording pair within the set. These two lists were combined to create a final list to assign each participant to Set A or B and the order in which picture/audio recording pairs were displayed when each participant was recruited. 

After viewing all four picture/audio recording pairs of the actor anesthesiologists, participants completed a questionnaire. Participants were asked to rank each anesthesiologist in order of confidence, intelligence, and likelihood of choosing that anesthesiologist to care for their family member. Participants also chose the one anesthesiologist who seemed most like a leader. Participant demographic data including age, sex, and race/ethnicity were collected. 

Statistical analysis

The questionnaire items on confidence, intelligence, and care of family member were measured on a 1-4 ranking scale with 1 representing the best anesthesiologist and 4 representing the worst anesthesiologist for each measure. Leadership was measured as a binary indicator. A power analysis revealed that enrolling 300 patients would provide sufficient (>80%) power to detect a difference in ranking of a half-level or greater with a variance of one level for anesthesiologist race or sex on the response measures (confidence, intelligence, care of family member).

Multinominal logistic regression was applied to the outcomes for the ranking of actors on the measures of confidence, intelligence, and care of family member, and logistic regression to the outcome of leadership. These outcomes were correlated at an individual level due to multiple video viewing by each respondent. The generalized estimating equation method, an extension of the generalized linear model for correlated responses (continuous, binary, or discrete), was used to account for correlated outcomes in this study. 

The main covariates of interest were the actor anesthesiologists’ race and sex. We adjusted for respondents’ age, sex, and race. We tested for interactions between actor anesthesiologists’ race and respondents’ race. We also tested for other two-way interactions between actor and respondent characteristics (e.g., between anesthesiologists’ sex and respondents’ race), but they were not statistically significant and thus excluded from the final models. We tested for differences between randomization groups (Set A and Set B image/audio pairings), but did not identify differences in either patient characteristics (i.e., randomization created balanced groups) or in observed effects (i.e., effects were consistent in both groups). The image/audio pairing set was thus not included in the final models. Final models were chosen to include statistically-significant interactions and adjust for respondent characteristics even if not significantly associated with the outcome. A two-sided test with a P-value <0.0167 that accounts for multiple comparisons was considered statistically significant. All data analyses were performed using SAS, version 9.4 (SAS Institute, Inc., USA), particularly using Proc GENMOD for the generalized estimating equation modeling [11]. The marginal effects of anesthesiologist- and respondent-specific characteristics on the outcomes of interest were estimated and reported.

## Results

Three hundred forty-six participants were approached about possible enrollment in the study (Figure 1). Forty-six total patients were excluded as they either did not meet inclusion criteria (n=9) or declined to participate (n=37). Three hundred participants viewed the 4 image/audio pairs and completed the questionnaire. Table 1 depicts the demographics of the participants randomized to image/audio Set A and Set B. There was no observed difference in age (p=0.71), sex (p=0.94), or race/ethnicity (p=0.15) of the participants randomized to each image/audio set. Seventeen participants’ responses were excluded from the final analysis for not following directions (e.g., ranked every actor the same on each measure, did not provide rankings for select questions). The demographics of the 17 excluded participants were similar to the overall sample (data not shown).

**Table 1:** Participant Demographics Demographic data for the two participant groups randomized to image/audio pair Set A and Set B.

PARTICIPANT DEMOGRAPHICS				
		Set A (n = 152)	Set B (n = 148)	Total (n = 300)
Age	Range	18 - 81	18 - 85	18 - 85
	Mean (SD)	55 (15)	54 (17)	55 (16)
Sex	Male (%)	60 (39)	59 (40)	119 (40)
	Female (%)	92 (61)	89 (60)	181 (60)
Race	White (%)	129 (85)	116 (78)	245 (82)
	Black (%)	16 (10)	28 (19)	44 (15)
	Hispanic (%)	4 (3)	1 (1)	5 (2)
	Asian (%)	3 (2)	0 (0)	3 (1)
	Other (%)	0 (0)	3 (2)	3 (1)

Results of the questionnaire data for the ranked primary outcomes (confidence, intelligence, and likelihood of choosing the anesthesiologist to care for their family member) are provided in Table 2. Lower scores (scores closer to 1) indicate the participants ranked that anesthesiologist more highly as the anesthesiologists were ranked on a 1-4 scale with 1 representing the best anesthesiologist for each measure.

**Table 2:** Ranked Outcome Measure Results Results (mean and median) for the ranked outcome measures: confidence, intelligence, and likelihood of choosing the anesthesiologist to care for a family member. Lower scores represent the anesthesiologist was ranked more highly on a scale of one to four (with one representing the best anesthesiologist for that measure). Values are expressed as number of responses (n), mean (95% confidence interval), and median [25th, 75th percentile].

	CONFIDENCE			INTELLIGENCE			CARE FOR FAMILY MEMBER		
	n	Mean (95% CI)	Median [25^th^, 75^th^]	n	Mean (95% CI)	Median [25^th^, 75^th^]	n	Mean (95% CI)	Median [25^th^, 75^th^]
Black female	280	2·4 (2·2, 2·5)	2 [1, 4]	280	2·2 (2·1, 2·4)	2 [1, 3]	283	2·2 (2·1, 2·3)	2 [1, 3]
Black male	282	2·4 (2·3, 2·5)	2 [2, 3]	282	2·4 (2·3, 2·5)	2 [1, 3]	279	2·4 (2·3, 2·6)	2 [2, 3]
White female	279	2·6 (2·5, 2·7)	3 [2, 4]	278	2·6 (2·5, 2·7)	3 [2, 4]	281	2·5 (2·4, 2·7)	3 [2, 4]
White male	281	2·6 (2·5, 2·8)	3 [2, 4]	278	2·8 (2·7, 2·9)	3 [2, 4]	281	2·8 (2·7, 3·0)	3 [2, 4]

Confidence

Anesthesiologists' race was associated with a difference in patient ranking of confidence (Table 3). Black anesthesiologists had greater odds of being ranked more confident than white anesthesiologists (odds ratio, 1.45; 95% CI, 1.10 to 1.89; P = 0.008). The sex of the anesthesiologist was not associated with differences in patient ranking of the actor anesthesiologists’ confidence (odds ratio, 1.10; 95% CI, 0.89 to 1.37; P = 0.380). Patient participant age, sex, and race were not associated with differences in patient ranking of confidence.

**Table 3:** Odds Ratios of Participant Responses for Each Outcome Measure Odds ratios with 95% confidence intervals for race (black versus white anesthesiologist) and sex (female versus male anesthesiologist) for the ranked outcome measures (confidence, intelligence, and likelihood of choosing the anesthesiologist to care for a family member) and binary outcome (leadership) adjusted for age, sex, and ethnicity of respondents.

Outcome Measure		Odds Ratio	95% Confidence Interval		
			Lower	Upper	P value
CONFIDENCE	Actor race (black), all respondents	1·45	1·10	1·89	0·008
	Actor sex (female), all respondents	1·10	0·89	1·37	0·380
	Respondent age	1·00	0·99	1·00	0·261
	Respondent sex (female)	0·98	0·95	1·01	0·213
	Respondent race	0·99	0·97	1·01	0·198
INTELLIGENCE	Actor race (black), white respondents	2·08	1·54	2·81	<0·001
	Actor race (black), nonwhite respondents	0·99	0·58	1·68	0·968
	Actor sex (female), all respondents	1·36	1·08	1·71	0·009
	Respondent age	1·00	0·99	1·00	0·896
	Respondent sex (female)	0·99	0·96	1·02	0·695
CARE FOR FAMILY MEMBER	Actor race (black), white respondents	2·26	1·66	3·08	<0·001
	Actor race (black), nonwhite respondents	0·78	0·44	1·39	0·404
	Actor sex (female), all respondents	1·58	1·27	1·97	<0·001
	Respondent age	1·00	0·99	1·00	0·653
	Respondent sex (female)	1·00	0·97	1·04	0·862
LEADERSHIP	Actor race (black), all respondents	2·06	1·50	2·84	<0·001
	Actor sex (female), all respondents	1·31	0·95	1·81	0·095
	Respondent age	1·00	0·99	1·00	0·560
	Respondent sex (female)	1·03	0·97	1·09	0·306
	Respondent race (nonwhite)	1·06	1·02	1·11	0·005

Intelligence

Both, race and sex of the anesthesiologist were associated with differences in patient ranking of intelligence (Table 3). Black anesthesiologists had greater odds of being ranked more intelligent than white anesthesiologists (odds ratio, 2.08; 95% CI, 1.54 to 2.81; P<0.001) among white respondents. The race of the anesthesiologist was not associated with a difference in the ranking of intelligence among nonwhite participants (odds ratio, 0.99; 95% CI, 0.58 to 1.68; P=0.968). Female anesthesiologists had greater odds of being ranked more intelligent than male anesthesiologists (odds ratio, 1.36; 95% CI, 1.08 to 1.71; P=0.009). Participant age and sex were not associated with differences in patient ranking of anesthesiologist intelligence.

Care of family member

Both race and sex of the anesthesiologist were associated with differences in ranking the anesthesiologist they would most prefer care for their family member (Table 3). Among white respondents, black anesthesiologists had greater odds of being ranked higher as the anesthesiologist they would choose to care for their family member (odds ratio, 2.26; 95% CI, 1.66 to 3.08; P<0.001). The race of the anesthesiologist was not associated with a difference in ranking among nonwhite participants on this measure (odds ratio, 0.78; 95% CI, 0.44 to 1.39; P=0.404). Female anesthesiologists had greater odds of being ranked higher on the care of family member measure (odds ratio, 1.58; 95% CI, 1.27 to 1.97; P < 0.001). Participant age and sex were not associated with differences in ranking.

Leadership

Of the 276 participants who responded to the leadership measure, 96 (35%) selected the black female anesthesiologist, 79 (29%) selected the black male, 56 (20%) selected the white female, and 45 (16%) selected the white male anesthesiologist (Table 4). Black anesthesiologists had greater odds of being chosen as the anesthesiologist who seemed most like a leader (odds ratio, 2.06; 95% CI, 1.50 to 2.84; P < 0.001; Table 3). Female anesthesiologists had similar odds of being chosen as the anesthesiologist who seemed most like a leader (odds ratio, 1.31; 95% CI, 0.95 to 1.81; P = 0.095). Participant age and sex were not associated with differences in participant responses. Participant leadership responses were different based on participant race (odds ratio, 1.06; 95% CI, 1.02 to 1.11; P=0.005); however, the selection differences between non-white and white respondents were not based on the sex or race of the actor anesthesiologist (rather, due to an unmeasured characteristic of the anesthesiologists). 

**Table 4:** Leadership Outcome Measure Results for number and proportion of patients who selected each anesthesiologist as seeming most like a leader.

	LEADERSHIP	
	# of responses (n=276)	Percent of total responses (%)
Black female	96	35
Black male	79	29
White female	56	20
White male	45	16

## Discussion

Contrary to our hypothesis, patients preferred black anesthesiologists to white anesthesiologists across all measures: confidence, intelligence, likelihood of choosing the anesthesiologist, and leadership. Female anesthesiologists were more likely to be ranked higher on the measures of intelligence and likelihood of choosing the anesthesiologist to care for their family member compared to male anesthesiologists. Responses were consistent between both sets of audio recordings, suggesting that patient preferences are strongly biased by visual factors. These results support our previous work [9,10] by demonstrating that patients do have preferences for certain anesthesiologists after a review of simulated preoperative encounters with actor anesthesiologists. However, our data in this study were likely impacted significantly by social desirability bias given current events and increasing societal awareness.

While the association between physicians’ implicit biases and racial disparities in healthcare delivery is well documented [12-14], patients’ own implicit or explicit biases may also affect the physician-patient relationship though this is less often discussed [15]. Directly in contrast to our hypothesis, patients ranked black physicians more highly than white physicians on multiple measures of competence and leadership ability. Interestingly, several participants in our study declined to complete all questions on the study questionnaire or ranked all physician-actors the same (n=17) with many stating they did not feel comfortable ranking the physician-actors, particularly on the measure of intelligence. This may suggest an increased awareness of and sensitivity to racism. Alternatively (or additionally), this may reflect social desirability bias in which respondents strive to portray themselves favorably when they are directly observed by someone who they perceive may pass judgement. This phenomenon was demonstrated in a study examining the expression of anti-black prejudice by white Americans, wherein negative views were expressed less frequently during face-to-face interviews with an examiner compared to responses collected via computer-assisted interview [16]. Despite using an anonymous questionnaire in our study, respondents may not have answered truthfully or declined to complete the questionnaire to avoid judgement due in part to the questionnaire being distributed in person by one of the researchers (E.K.) rather than electronically.

Our data demonstrating patient preferences for black anesthesiologists on multiple measures of competence were unexpected though not entirely unprecedented. As our participants were comprised primarily of white patients, we predicted white anesthesiologists would be ranked more favorably not only due to racial/ethnic concordance, but also due to the pervasive impact of racism within both society and the field of medicine. Much of the available literature in this area focuses on racial/ethnic concordance between the patient and the physician and the impact on health-related outcomes among these studies have demonstrated heterogeneous results [17-19]. A comprehensive review of 27 studies published between 1980-2008 found that patient-provider race-concordance was associated with positive health outcomes for minorities in 9 (33%), no association with health outcomes in 8 (30%), and mixed results in 10 (37%) of the studies [18]. 

In addition to the potential impact of social desirability bias and patient awareness as discussed above, our study has several limitations. While we attempted to control for many factors when recruiting, photographing, and recording the actor anesthesiologists (e.g., age, body language, voice characteristics), there are likely unmeasured factors (e.g., perceived compassion) that contributed to the differences detected in participant ranking of the anesthesiologists and were likely magnified by using one actor to represent an entire race-sex group. Additionally, as all actors were approximately the same age (in their 30s), these data may reflect perceptions involving early career-appearing anesthesiologists and may not be generalizable to mid- or late career-appearing anesthesiologists. Our study also involved a patient population that was predominantly white and exclusively English-speaking. Thus, our results are not generalizable to regions or communities with more diversity. Patient perceptions may be influenced by other unmeasured variables (e.g., baseline anxiety or depression) that were not collected in our study. Finally, by not allowing for open-ended responses or using semi-structured interviews, we are limited in the interpretation of our data and cannot fully understand the reasoning behind our patients’ questionnaire responses.

## Conclusions

Contrary to our hypothesis, patients ranked black physicians more highly on multiple competence and leadership quality metrics in a simulated preoperative encounter. Our data likely highlight the role social desirability bias and current events in society may play in studies of racial disparities within medicine. While we are hopeful that our study does reflect shifting perceptions of physicians based on race and a greater appreciation that a person’s skin color does not provide information on the competence of the physician, continued investigation in this area and support of a diversified workforce that reflects the patient population remain priorities.

## Figures and Tables

**Figure 2:**
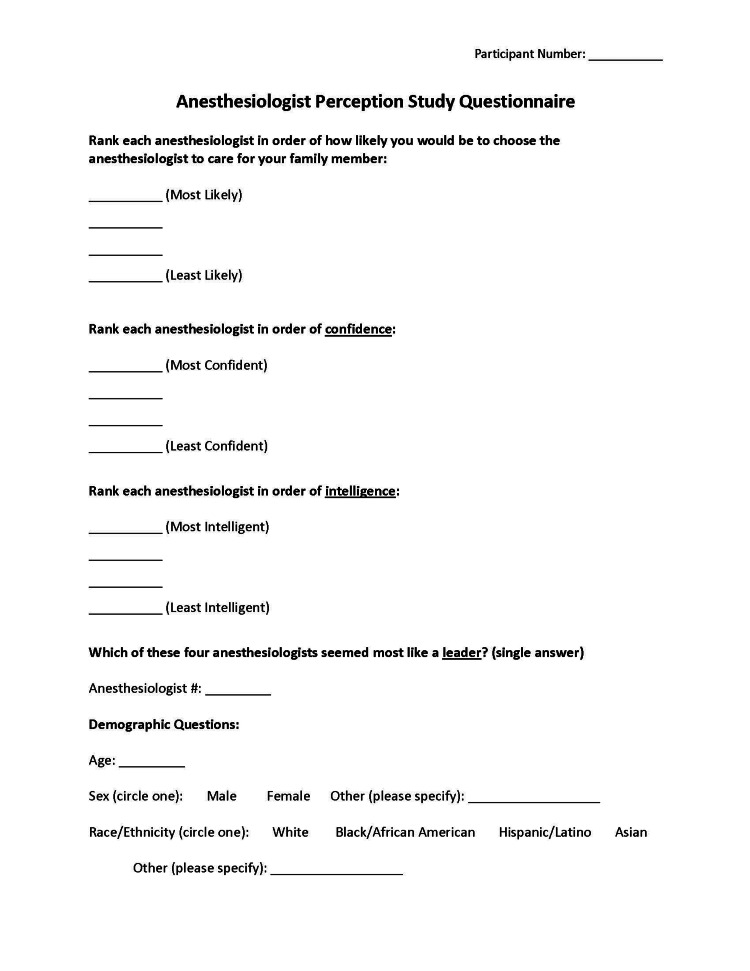
Study Questionnaire
